# Monitoring of radioactive cesium in wild boars captured inside the difficult-to-return zone in Fukushima Prefecture over a 5-year period

**DOI:** 10.1038/s41598-022-08444-1

**Published:** 2022-04-19

**Authors:** Rie Saito, Reiko Kumada, Kenji Inami, Kousuke Kanda, Masahiko Kabeya, Masanori Tamaoki, Yui Nemoto

**Affiliations:** 1 Fukushima Prefectural Centre for Environmental Creation, 10-2 Fukasaku, Miharu-machi, Fukushima 963-7700 Japan; 2 Fukushima Regional Collaborative Research Center, National Institute for Environmental Studies, 10-2 Fukasaku, Miharu-machi, Fukushima 963-7700 Japan; 3 Wildlife Symbiosis Centre, 67 Nagakubo, Tamai, Ootama Village, Fukushima 969-1302 Japan; 4 Biodiversity Division, National Institute for Environmental Studies, 16-2 Onogawa, Tsukuba, Ibaraki 305-8506 Japan; 5 Okutama Practice Forest, Tokyo University of Agriculture, Hikawa 2137, Okutama, Tokyo 198-0212 Japan

**Keywords:** Ecology, Environmental sciences

## Abstract

Following the Fukushima Daiichi Nuclear Power Plant accident in 2011, tissue samples from wild boar (*Sus scrofa*) outside the evacuation zone (difficult-to-return zone, DRZ) tended to show high activity concentrations of cesium-137 (^137^Cs). Understanding the ^137^Cs dynamics of wild boar populations inside the DRZ is necessary because they affect ^137^Cs dynamics and wild boar management in areas outside the DRZ. Since few detailed, long-term studies have been conducted inside the DRZ, we measured ^137^Cs activity concentrations in 221 wild boar muscle samples obtained from wild boar caught inside the DRZ and surrounding areas over a 5-year period. Our results showed that the ^137^Cs activity concentration in wild boar from inside the DRZ were higher than those in wild boar outside this zone. No significant difference was observed between muscle and soil ^137^Cs levels, but significant correlations were observed between muscle ^137^Cs activity concentrations and body length and body weight in the low-activity-concentration season, but not between all seasons and the high-activity-concentration seasons. It is considered that the size effects observed during the low-activity-concentration season may be due to factors related to metabolism and changes in food habit. This is the first long-term survey of ^137^Cs in wild boar inside the DRZ.

## Introduction

The Fukushima Daiichi Nuclear Power Plant (hereafter, FDNPP) accident occurred as a result of the Great East Japan Earthquake in March 2011. Due to large amounts of artificial radioactive materials being released as a result of the FDNPP accident, the area within a 20-km radius around the FDNPP was immediately designated as the Warning area to prevent radiation exposure^1^. In addition, areas with radiation dose rates above 20 mSv/year were designated as Planned Evacuation Zones and access to these areas was restricted^1^. Subsequently, three evacuation zones—the difficult-to-return zone (hereafter, DRZ), the Restricted Residence Zone, and the Evacuation Order Cancellation Preparation Zone––were established based on predicted radiation doses to humans. Even though more than 10 years have passed since the accident, access to the DRZ is still restricted and previous residents of the DRZ have been prevented from returning to their homes due to the high radiation dose rates (> 50 mSv/year)^2^.

Large amounts of radionuclides were released from the reactors at the time of the FDNPP accident^3^. Of these, concerns surrounding the long-term accumulation of cesium-137 (^137^Cs) in wildlife have been raised due to its relatively long physical half-life (30 year) and high bioavailability (i.e., absorption and transfer rates), as its chemical characteristics are similar to those of the monovalent cation potassium^4,5^. A high proportion of the radiocaesium released exists in an ionic state immediately after deposition in the soil, and then, after physical and chemical changes in the soil following deposition, radiocaesium binds to soil particles over time and becomes less easily dissolvable^6^. Such a physicochemical fraction of radiocaesium in the environment can also affect radiocaesium intake in wildlife [e.g.,^5,7^]. After the FDNPP accident, numerous studies reported that ^137^Cs was present in many wildlife species, and extensive studies have been conducted on ^137^Cs dynamics in organisms distributed in and around Fukushima (e.g., insects^8^; amphibians^9^; fishes^10–12^; birds^13^; and mammals^14–21^).

Since the FDNPP accident in 2011, the government of Fukushima Prefecture has conducted radioactive monitoring surveys of game meat, such as meat from wild boars (*Sus scrofa)* and Asian black bears (*Ursus thibetanus*)^22^. The results have shown that radiocaesium contamination differs between these species, and that wild boars have higher radiocaesium levels^16,22^. However, these studies did not examine the DRZ, which has high levels of radionuclide contamination. A previous study showed that there was a positive correlation between the ^137^Cs activity concentration in wild boar muscle and ^137^Cs deposition on soil at the sites where the wild boars were captured^16^. ^137^Cs deposition on soil in the DRZ was high after the accident and most of the area has not yet been decontaminated. Therefore, the wild boars in the DRZ likely have high levels of ^137^Cs contamination. In addition, ^137^Cs activity concentration levels of wild boars remain high and constant over the several decades after the Chernobyl nuclear power plant accident (hereafter, Chernobyl accident)^15,23^. According to the long-term monitoring, radiocaesium activity concentration of wild boars observed little to no decline or even a slight increase in activities in some case^15^. In case of the FDNPP accident, the long-term contamination in wild boars is of great concern.

After the FDNPP accident, restrictions were placed on the shipment of foodstuffs throughout Fukushima Prefecture and on the consumption of wild boar meat in parts of the prefecture^22^. Removal of these restrictions is difficult because several wild boar meat samples have been found to contain radionuclide activity concentrations that exceed acceptable levels for consumption (i.e., samples that have total radionuclide (^134^Cs and ^137^Cs) levels that exceed the 100 Bq/kg limit prescribed by Japanese food standards).

Wild boars are an important game species in the region, and they cause serious agricultural damage. In recent years, agricultural damage by wild boars has increased and become more widespread due to the marked increase in the wild boar population, which in turn is attributed to the decrease in human activity in rural areas, the increase in deserted arable land, and the decrease in hunting pressure as hunters age and decrease in number^24,25^. In response to this increase in wild boar numbers, prefectures and municipalities have implemented control measures to capture wild boars and reduce the damage that they cause.

A unique problem for the management of wild boar in Fukushima Prefecture is that the motivation among hunters to hunt wild boar has decreased due to the restrictions imposed on the utilization of radionuclide-contaminated wild boar meat^26^. In addition, there is concern that wild boars in human settlements will transport high activity concentrations of radionuclides beyond the boundaries of the DRZ^26^. Of particular concern in the management of wild boar in the DRZ is that a decrease in human activities, such as agricultural activities, in the zone will result in both range expansion and population increases of wild boars in the region^26^. Consequently, clarifying the ^137^Cs activity concentration dynamics in wild boar inside the DRZ is considered necessary because this factor appears to be closely related to ^137^Cs dynamics and wild boar management in areas outside the DRZ.

Few studies on ^137^Cs activity concentrations in wild boars inside the DRZ and the surrounding areas have been conducted; these studies include the results of 1-year trends in Tomioka town^19^, and a comparison of methods used to examine aggregated transfer factors (T_ag_: radionuclide activity concentration in muscle/radionuclide deposition in soil, m^2^/kg)^27^. However, no detailed reports based on long-term or extensive sampling have been conducted inside the DRZ.

**Figure 1:**
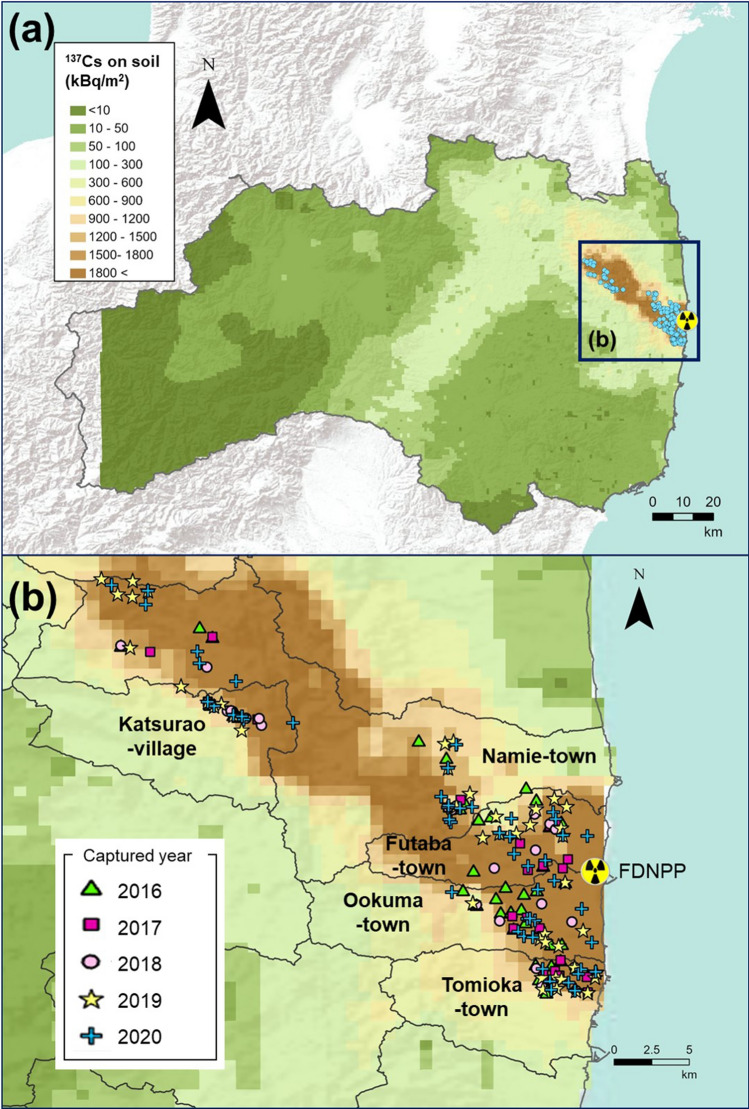
Sites where wild boars were captured in the difficult-to-return zone (DRZ) (**a**) Fukushima Prefecture, (**b**) DRZ and surrounding area (including the Specified Reconstruction and Revitalization Base areas). Symbols on the map indicate the sites where wild boars were captured. Differences in the shape and color of symbols indicate differences in the year of capture. This original map was created using ArcGIS Pro 3.1.6 (https://www.esri.com/en-us/arcgis/products/arcgis-pro/overview). The map of Fukushima Prefecture was obtained from the Ministry of Land, Infrastructure, Transport and Tourism (MLIT) of Japan (http://nlftp.mlit.go.jp/ksj/), and ^137^Cs deposition on soil was based on from the 5th airborne monitoring survey (https://emdb.jaea.go.jp/emdb/en/portals/b1020201/) by the Japan Atomic Energy Agency (JAEA).

In this study, we conducted a monitoring survey of ^137^Cs activity concentrations in muscle samples collected from wild boar inside the DRZ and surrounding areas (Fig. 1) over a 5-year period.

## Results

### Comparison of ^137^Cs activity concentrations in wild boar muscles collected from wild boars captured inside and outside the DRZ

**Figure 2:**
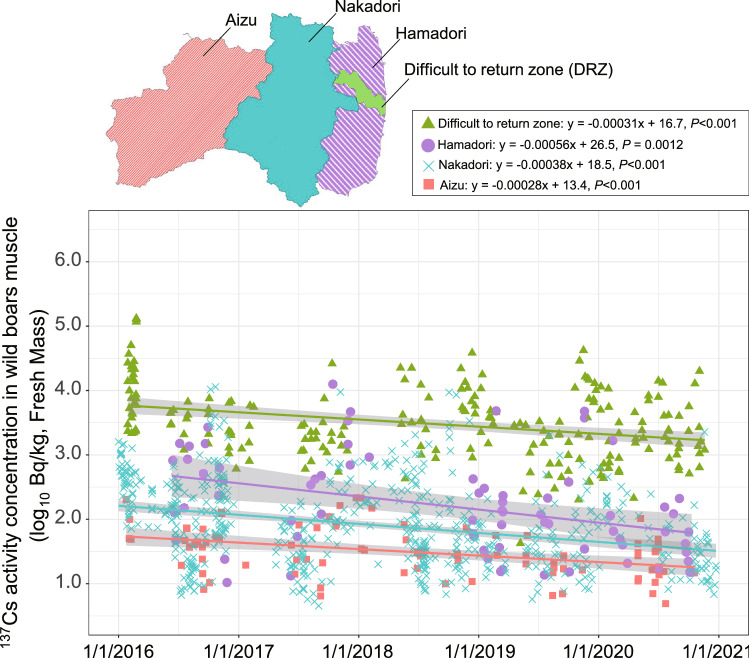
Temporal changes in ^137^Cs activity concentrations in wild boar muscle samples collected inside the difficult-to-return zone (DRZ) and three regions outside the DRZ (Hamadori, Nakadori and Aizu). The map shows each region using different colors. The DRZ (10 March in 2020) on the map includes the specific reconstruction areas in which restrictions on entry have been lifted. The simple linear regression equation (each line) and the 95% confidence interval about the regression line (grey shaded area) are shown in the figure. The original map was created using ArcGIS Pro 3.1.6 (https://www.esri.com/en-us/arcgis/products/arcgis-pro/overview). The map of Fukushima Prefecture was obtained from the Ministry of Land, Infrastructure, Transport and Tourism (MLIT) of Japan (http://nlftp.mlit.go.jp/ksj/) and area of difficult to return zone (DRZ) was created based on conceptual diagram of evacuation area on March 10 in 2020 (https://www.pref.fukushima.lg.jp/site/portal/cat01-more.html).

**Figure 3:**
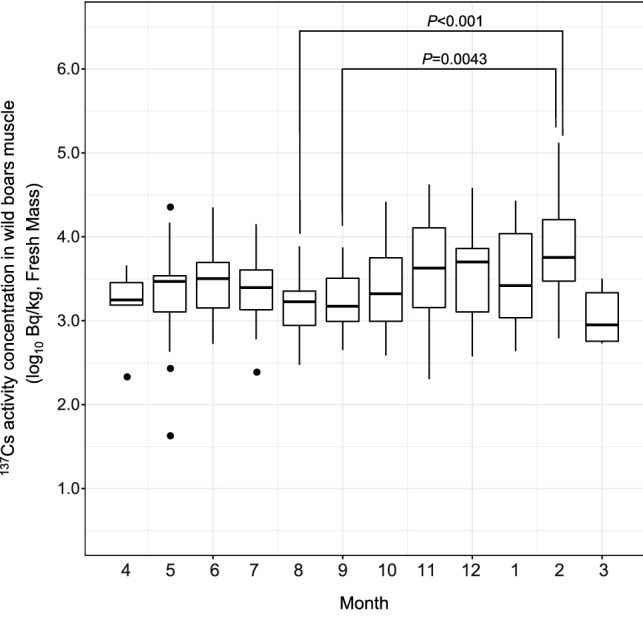
Seasonal variation in ^137^Cs activity concentration muscle samples from wild boar inside the difficult-to-return-zone (DRZ). The upper part of the box plot shows the 75th percentile and the lower part shows the 25th percentile. The horizontal lines in the box indicate the median value. The whiskers above and below the boxes indicate the maximum and minimum values, respectively. Black dots indicate outliers. The *P* values were calculated using the Steel–Dwass test (*P* < 0.01) between each month.

The ^137^Cs activity concentrations for all muscle samples from wild boars captured inside the DRZ exceeded detection limits, with levels greater than 100,000 Bq/ kg (FM) obtained for three samples collected in 2016 (Fig. 2, Table 1). The detection range was from 42 to 132,210 Bq/kg (FM), indicating that there was a large variation among individuals (Fig. 2, Tables 1, 2). Significant correlations were found between captured date and the ^137^Cs activity concentration in in all four regions (i.e., inside the DRZ and three regions outside the DRZ (Hamadori, Nakadori, and Aizu)) (Fig. 2). The ^137^Cs activity concentrations obtained from wild boar muscle samples collected inside the DRZ tended to be higher than those collected outside the DRZ (Fig. 2). Effective half-life (T_eff_) in each region were: 5.1 year (DRZ), 3.0 years (Hamadori), 4.3 years (Nakadori) and 5.5 years (Aizu). In addition, ^137^Cs activity concentrations in meat samples varied significantly by sampling month (Fig. 3, Table 2, Kruskal–Wallis test, df = 11, χ^2^ = 35.18, *P* < 0.001). Specifically, significant differences were observed between February in the high-activity-concentration season and August and September in the low-activity-concentration season (Fig. 3, Table 2, Steel–Dwass’ test, *P* < 0.01).

**Table 1:** ^137^Cs activity concentration in wild boars muscle of each year in difficult to return zone.

Year	Mean (Bq/kg, FM)	Max (Bq/kg, FM)	Minimun (Bq/kg, FM)	Numver of indivicuals (N)
2016	15,028	132,210	608	56
2017	3030	26,110	542	29
2018	8303	38,385	427	30
2019	5180	42,018	42	52
2020	3508	20,010	213	54

**Table 2:** ^137^Cs activity concentration in wild boars muscle of each month in difficult to return zone.

Month	Mean (Bq/kg, FM)	Max (Bq/kg, FM)	Minimun (Bq/kg, FM)	Numver of indivicuals (N)
1	6797	26,909	434	16
2	17,289	132,210	620	44
3	1463	3165	538	5
4	2189	4566	213	5
5	4883	22,490	42	12
6	4992	22,351	531	13
7	3892	14,138	243	25
8	1917	7719	298	18
9	2208	7479	448	16
10	4842	26,110	386	17
11	8730	42,018	201	26
12	6236	38,385	377	24

### Relationships among ^137^Cs activity concentration in muscle and ^137^Cs deposition on soil, body length and body weight

**Figure 4:**
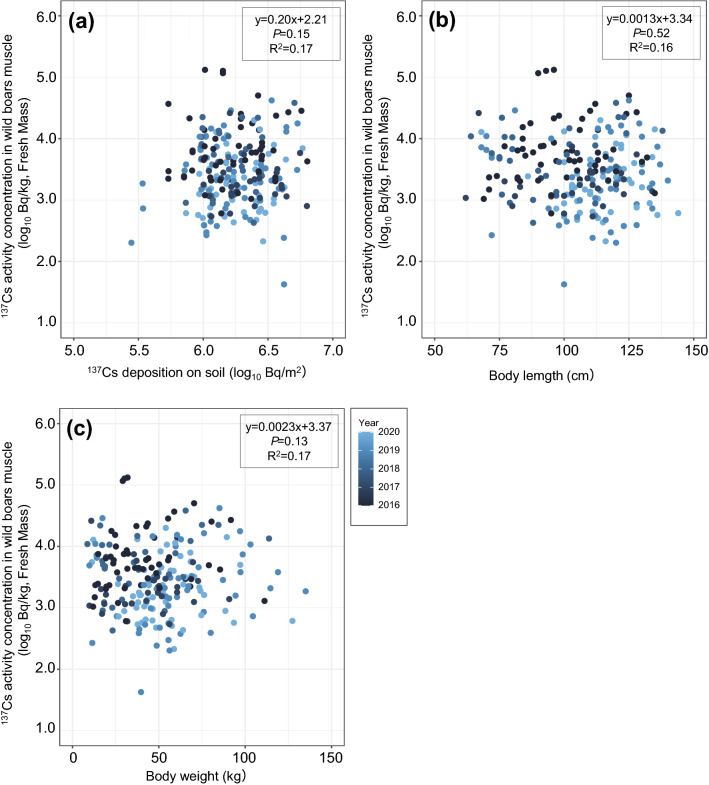
Relationships between ^137^Cs activity concentration in wild boar muscle samples collected in all seasons and (**a**) ^137^Cs deposition on soil (log_10_ Bq/m^2^), (**b**) body length (cm), and (**c**) body weight (kg) inside the difficult-to-return zone (DRZ). Results of the regression analysis are based on mixed linear models using years as random variables and the adjusted coefficient of determination (R^2^) are shown in the figures.

**Figure 5:**
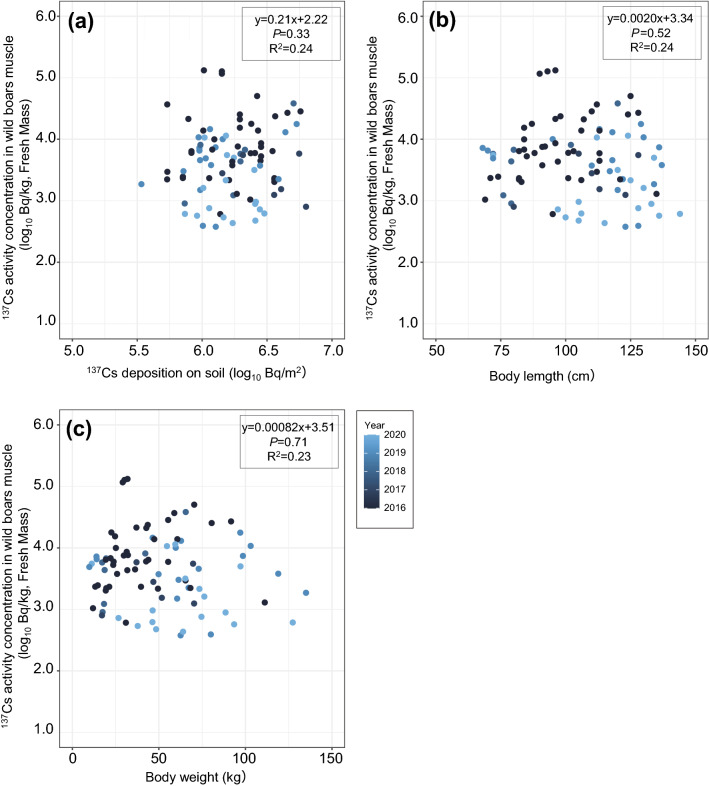
Relationships between ^137^Cs activity concentration of wild boar muscle samples collected in the high-activity-concentration season (December to March) from inside the difficult-to-return zone (DRZ) and (**a**)^137^Cs deposition on soil (log_10_ Bq/m^2^), (**b**) body length (cm), and (**c**) body weight (kg). Results of the regression analysis are based on mixed linear models using years as random variables and the adjusted coefficient of determination (R^2^) are shown in the figures.

**Figure 6:**
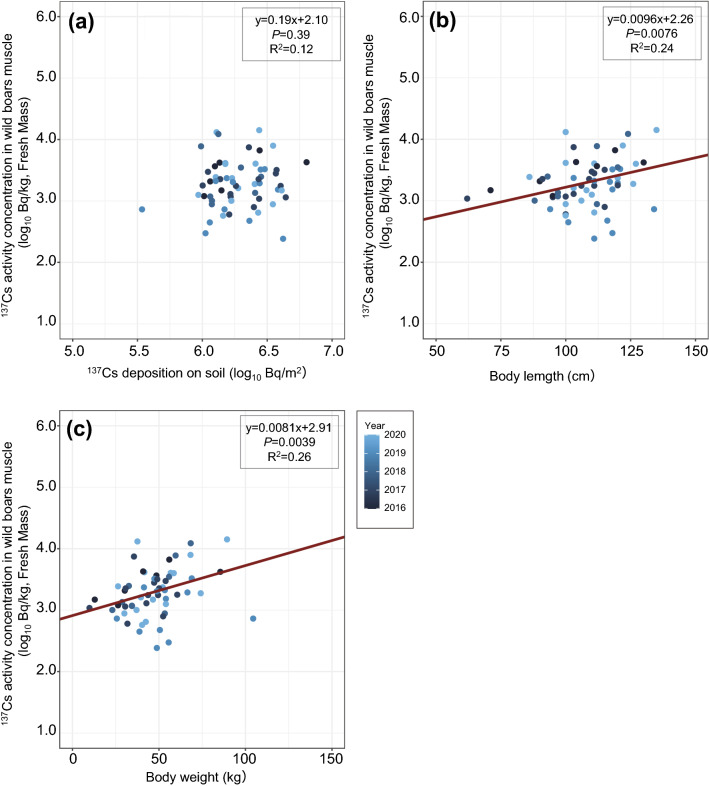
Relationships between the ^137^Cs activity concentration of wild boar muscle samples collected in the low-activity-concentration season (July to September) from inside the difficult-to-return zone (DRZ) and (**a**) ^137^Cs deposition on soil (log_10_ Bq/m^2^), (**b**) body length (cm), and (**c**) body weight (kg). Results of the regression analysis are based on mixed linear models using years as random variables and the adjusted coefficient of determination (R^2^) are shown in the figures. The linear regression equation (red line) is shown only when a significant difference between the two variables was observed.

From the results of the regression analysis in all seasons and the high-activity-concentration season, no significant differences were observed among the ^137^Cs activity concentration in muscle and ^137^Cs deposition on soil, body length and body weight (Figs. 4, 5). Also, in the low-activity-concentration season, no significant differences were observed between the ^137^Cs activity concentration in muscle and ^137^Cs deposition on soil, but significant correlations were observed among the ^137^Cs activity concentration in muscle and body length and body weight (Fig. 6).

## Discussion

The ^137^Cs activity concentrations in wild boar muscle samples from inside the DRZ were higher than those from outside the DRZ. In addition, a large variation was observed among individuals, with differences of several orders of magnitude recorded (Fig. 2). In our study, the range of T_eff_ was 3.0–5.5 years. T_eff_ of wild boars affected by the Chernobyl accident were reported: 7.8 years at Bavaria^15,28^, 1.7 years at southern Germany under 3 years monitoring^15^, 11.7 years at “alienation zone’’ and 92 years at ‘‘periodic control zone’’ in the Chernobyl exclusion zone^15^. Our results of T_eff_ were less than physical half-lives and not so different from the past reports in the case of Chernobyl [e.g., case of Bavaria, southern Germany]. But our results are based on only 6 years of monitoring the ^137^Cs activity concentration. Since a portion of the cases reported little to no decline or even a slight increase in radiocaesium activity concentrations of wild boars after the Chernobyl accident^15^, we need to continue to focus on long-term fluctuations regarding the FDNPP accident. In our study, the ^137^Cs activity concentration in wild boar muscles tended to be low in August and September in the summer season and high in February in the winter season inside the DRZ, indicating that our results corroborate the findings of a previous study^16^. In Tomioka town, which includes areas inside the DRZ, the ^137^Cs activity concentration in wild boar muscle was previously reported to vary significantly between different months^19^.

No significant differences were observed between the ^137^Cs activity concentrations in muscle and ^137^Cs deposition on soil inside the DRZ in any of the time periods examined (i.e., all seasons, high-activity-concentration and low-activity-concentration seasons) (Figs. 4, 5, 6). ^137^Cs deposition on soil tended to be higher in the DRZ than outside the DRZ; however, decontamination efforts have subsequently been initiated in areas close to and inside the DRZ (e.g., Specified Reconstruction and Revitalization Base). Therefore, the distribution of ^137^Cs on soil is highly heterogeneous in the region. This heterogeneity might account for the absence of any relationships between muscle ^137^Cs activity concentrations and ^137^Cs deposition on soil in this area. However, a previous study also reported that muscle ^137^Cs activity concentrations in wild boars was highly variable, even when wild boars were captured in areas with similar ^137^Cs soil deposition levels^16^. In addition, it is considered that the evaluation of the radiocaesium contamination level in animal species based on soil deposition (i.e., T_ag_ values) is inappropriate for animal species with large home ranges such as wild boars^27^. Similar findings were reported in moose after the Chernobyl accident, where there was a weak correlation between ^137^Cs soil deposition and the ^137^Cs activity concentration in muscle^29^. In that study, the authors proposed that the annual fluctuations in the T_ag_ values observed in moose may have been attributed to various ecological factors, such as differences in food selection or habitat use^29^. Seasonal variation of food resources and plants organs (e.g., herbs and roots) have been observed in wild boar diet^30,31^ and it seems that food preference/selection occurs at an individual level. In addition, ^137^Cs activity concentration in food resources of wild boars varies widely depending on ^137^Cs contamination levels in the environment (e.g., ^137^Cs deposition on soil), plant diversity and the unique distribution of ^137^Cs contamination by plant species (e.g., species-specific levels of ^137^Cs contamination found in roots, leaves, berries, etc.). Along with the consumption of food resources the ingestion of soil affects the increase of ^137^Cs level in wild boar muscle is unclear^7^. In this study, we considered that the variations observed in the ^137^Cs activity concentrations in the muscle of wild boars may be more strongly affected by ecological factors, such as food habits and migration than to ^137^Cs soil deposition at capture site.

In some fish species, a size effect in which there is a tendency for radiocaesium activity concentration in the body to accumulate as a function of increased body size and/or weight has been reported^11^. The factors affecting this size effect were considered to be related to ontogenetic changes in food habits and/or the physiological ability to retain radiocaesium during growth^11,32^. In our study, a significant positive relationship was observed between muscle ^137^Cs activity concentration and both body length and weight of wild boars during the low-activity-concentration season, but not during all seasons and the high-activity-concentration season. Cui et al.^19^ found no significant correlation between radiocaesium activity concentration and the weight of the wild boars captured over the entire year in 2019–2020 in Tomioka town. A study on the mobility and home range size of wild boar reported that females with piglets have a smaller home range in the summer season than in the fall and parturitional season (i.e., spring)^33^. In France, the home range of wild boars in the hunting season are larger than in the summer season^34^. It is considered that the size effects observed during the low-activity-concentration season in our study may be due to factors related to metabolism and changes in food habits, because the movements and habitat shifts in wild boars are not as great as they are during the other seasons. However, since the activity, movement and home range of wild boars are influenced by numerous factors, such as food availability, population density and hunting pressure (e.g.^35,36^), it is important to clarify the relationships between muscle ^137^Cs activity concentration and the seasonal mobility of wild boars in areas including the DRZ in future studies.

Our study showed that the 5-year trend in the ^137^Cs activity concentration in the muscles of wild boar inside the DRZ was higher than that in wild boar from outside this area and confirmed the existence of seasonal variation in these activity concentrations and in the size effect of ^137^Cs accumulation in the low-activity-concentration season. After the Chernobyl accident, ^137^Cs activity concentrations in wild boars were higher than those in other wild animals^37^, with ^137^Cs levels showing long-term accumulation^15,23,38–40^. Consequently, we consider that long-term monitoring of radiocaesium dynamics should be undertaken in wild boars in areas affected by the FDNPP accident. Continued surveys focusing on the movement of wild boars inside the DRZ could facilitate the management of wild boar populations and the monitoring of radiocaesium dynamics in wild boars in areas outside the DRZ where high muscle ^137^Cs activity concentrations have occasionally been observed.

## Materials and methods

### Sample collection

We collected muscle tissue samples from 221 culled wild boar individuals that were trapped as part of a survey conducted by the Ministry of the Environment in Japan; “The habitat survey and capture of the wild animals project in and around the former restricted areas (areas within 20-km radius from Fukushima Daiichi NPP (2015–2017)” and “The habitat survey and capture of the wild birds and mammals project in and around the “difficult-to-return zone” in Fukushima (2018–2020)”. The capture of wild boar and the collection of muscle samples were conducted by the Japan Wildlife Research Center, which was commissioned to conduct these surveys. For all wild boar samples, the data collected included the GPS coordinates of the capture location, body length, body weight and sex. We used hind-leg muscle samples that were collected in five municipalities (Okuma, Katsurao, Tomioka, Namie, Futaba) from January in 2016 to November in 2020 (Fig. 1).

### Measurement of muscle ^137^Cs activity concentrations

The surface and any connective tissue were removed from the muscle block samples that were collected for analysis. Raw muscle samples were either minced, sliced, or freeze-dried for several days and then crushed using a bottle blender (Osaka Chemical Co., Ltd., Osaka, Japan). The samples were then placed in standard U-8 containers (100 ml, ⌀56 mm × 68 mm) and gamma-ray emitting radionuclides were measured using a germanium semiconductor detector (Canberra GC3018, Meriden, USA). The ^137^Cs activity concentration of freeze-dried muscle samples were calculated as fresh mass (hereinafter, “FM”). In addition, we used the ^137^Cs activity concentration data for wild boar muscles that were collected from outside the DRZ area as part of a wild animal monitoring survey conducted by the Fukushima prefectural government^22^. The results obtained for the samples that were collected outside the DRZ were divided into three groups based on the region of collection, i.e., Hamadori excluding the DRZ (hereafter, Hamadori), Nakadori and Aizu (Fig. 2); the ^137^Cs activity concentrations in these samples were compared against the results obtained for samples collected from wild boar inside the DRZ.

### Calculation of ^137^Cs deposited on soil

To examine whether there was any correlation between the ^137^Cs activity concentrations in wild boar muscles and ^137^Cs deposition on soil at the site of wild boar capture (Bq/m^2^), ^137^Cs activity concentrations were extracted from the ^137^Cs ground deposition open data map compiled by the Japan Atomic Energy Agency's (JAEA) 5th Airborne Monitoring Survey (JAEA, 2012)^41^ using ArcGIS Pro 3.1.6 (https://pro.arcgis.com/en/pro-app/latest/get-started/install-and-sign-in-to-arcgis-pro.htm). The ^137^Cs deposited on soil at each capture site was then estimated considering the decay rate over the number of days between the soil ^137^Cs measurements and the capture date of the wild boar using a physical half-life of ^137^Cs.

### Statistical analysis

To confirm the changes in the ^137^Cs activity concentration in wild boar muscle samples collected in four regions (i.e., inside the DRZ and three regions outside the DRZ (Hamadori, Nakadori, and Aizu)) over time, we performed a regression analysis of the relationship between ^137^Cs activity concentration in muscle and capture date (i.e., number of days to the capture date, with January 1, 1900 as the base date). Then, we excluded data that were below the detection limit [e.g.^42^]. The effective half-life (T_eff_) is one of the indicators of suitable measure to explain the behavior of radionuclides in various ecosystems and to predict future contamination levels^41^. T_eff_ is calculated below^15,43^:

$${1}/{\text{T}}_{{{\text{eff}}}} = { 1}/{\text{T}}_{{{\text{eco}}}} + { 1}/{\text{T}}_{{{\text{phys}}}}$$

Ecological half-life (T_eco_) of ^137^Cs was estimated for each region by T_eco_ = ln2/λ. Estimates of λ were obtained from the slope of the natural-log regression of ^137^Cs activity concentration versus time. T_phys_ was the physical half-life of ^137^Cs (30.2 year). Differences in the ^137^Cs activity concentration in wild boar muscle for each sampling month were evaluated using the Kruskal–Wallis test. Then, the Steel–Dwass test was performed to evaluate the difference in the mean values for each month. These statistical analyses were performed using R4.0.3 (https://www.r-project.org/).

We performed the regression analysis based on mixed linear models for wild boar datasets inside the DRZ. We used the ^137^Cs activity concentration in wild boar muscle as the response variable, and ^137^Cs deposition on soil, body length and body weight as explanatory variables. The capture year was used for a random factor. These analyses were performed using the JMP 13.2.1 software package (SAS, Cary, NC, USA). Because the ^137^Cs activity concentration in wild boar muscle exhibits seasonal variations^16^, we divided the data into three seasons, i.e., all seasons, high-activity-concentration season (December, January, February, March) and low-activity-concentration season (July, August, September). For all statistical analysis, we used the ^137^Cs activity concentration in muscle and ^137^Cs deposition on soil with log_10_ transformation.

## Data Availability

The datasets generated during and/or analyzed during the current study are available from the corresponding author on reasonable request.

## References

[1] Ministry of the Environment Government of Japan. *Designation of Evacuation Zone* (accessed 07 April 2021); https://www.env.go.jp/chemi/rhm/h29kisoshiryo/h29kiso-09-04-01.html. (**in Japanese**).

[2] Fukushima Prefectural Government, Japan. *About the Transition of Evacuation Zone * (accessed 07 April 2021); https://www.pref.fukushima.lg.jp/site/portal/cat01-more.html. (**in Japanese**).

[3] Chino M (2011). Preliminary estimation of release amounts of ^131^I and ^137^Cs accidentally discharged from the Fukushima Daiichi Nuclear Power Plant into the atmosphere. J. Nucl. Sci. Technol..

[4] Koarashi J, Atarashi-Andoh M, Takeuchi E, Nishimura S (2014). Topographic heterogeneity effect on the accumulation of Fukushima-derived radiocaesium on forest floor driven by biologically mediated processes. Sci. Rep..

[5] Saito R, Nemoto Y, Tsukada H (2020). Relationship between radiocaesium in muscle and physicochemical fractions of radiocaesium in the stomach of wild boar. Sci. Rep..

[6] Tsukada H (2012). From soil to agricultural-plants-transfer and distribution of radiocaesium. Kagaku (Chemistry)..

[7] Saito R, Tsukada H, Nanba K, Konoplev A, Wada T (2022). Chapter 23: Physicochemical fractions of radiocaesium in the stomach contents of wild boar and its transfer to muscle tissue. Behavior of Radionuclides in the Environment III.

[8] Ishii Y, Hayashi S, Takamura T (2017). Radiocaesium transfer in forest insect communities after the Fukushima Dai-ichi Nuclear Power Plant accident. PLoS ONE.

[9] Matsushima N, Ihara S, Takase M, Horiguchi T (2015). Assessment of radiocaesium contamination in frogs 18 months after the Fukushima Daiichi nuclear disaster. Sci. Rep..

[10] Ishii Y, Matsuzaki SS, Hayashi S (2020). Different factors determine ^137^Cs concentration factors of freshwater fish and aquatic organisms in lake and river ecosystems. J. Environ. Radioact..

[11] Wada T (2019). Strong contrast of cesium radioactivity between marine and freshwater fish in Fukushima. J. Environ. Radioact..

[12] Morishita D (2019). Spatial and seasonal variations of radiocaesium concentrations in an algae-grazing annual fish, ayu *Plecoglossus altivelis* collected from Fukushima Prefecture in 2014. Fish. Sci..

[13] Saito R, Kabeya M, Nemoto Y, Oomachi H (2019). Monitoring ^137^Cs concentrations in bird species occupying different ecological niches; game birds and raptors in Fukushima Prefecture. J. Environ. Radioact..

[14] Merz S, Shozugawa K, Steinhauser G (2015). Analysis of Japanese radionuclide monitoring data of food before and after the Fukushima nuclear accident. Environ. Sci. Technol..

[15] Steinhauser G, Saey PRJ (2016). ^137^Cs in the meat of wild boars: A comparison of the impacts of Chernobyl and Fukushima. J. Radioanal. Nucl. Chem..

[16] Nemoto Y, Saito R, Oomachi H (2018). Seasonal variation of caesium-137 concentration in Asian black bear (*Ursus thibetanus*) and wild boar (*Sus scrofa*) in Fukushima Prefecture, Japan. PLoS ONE.

[17] Nemoto Y, Oomachi H, Saito R, Kumada R, Sasaki M, Takatsuki S (2020). Effects of ^137^Cs contamination after the TEPCO Fukushima Dai-ichi Nuclear Power Station accident on food and habitat of wild boar in Fukushima Prefecture. J. Environ. Radioact..

[18] Saito R, Oomachi H, Nemoto Y, Osako M (2019). Estimation of the total amount of the radiocaesium in the wild boar in their body – each organs survey and incineration residue survey. J. Soc. Rem. Radioact. Contam. Environ..

[19] Cui L (2020). Radiocaesium concentrations in wild boars captured within 20 km of the Fukushima Daiichi Nuclear Power Plant. Sci. Rep..

[20] Tagami K, Howard BJ, Uchida S (2016). The time-dependent transfer factor of radiocaesium from soil to game animals in Japan after the Fukushima Dai-ichi nuclear accident. Environ. Sci. Technol..

[21] Fuma S (2016). Radiocaesium contamination of wild boars in Fukushima and surrounding regions after the Fukushima nuclear accident. Environ. Radioact..

[22] Fukushima Prefectural Government, Japan. Monitoring of Wild Animals. Accessed 7 Apr 2021. https://www.pref.fukushima.lg.jp/site/portal/wildlife-radiationmonitoring1.html. (**in Japanese**).

[23] Strebl F, Tataruch F (2007). Time trends (1986–2003) of radiocaesium transfer to roe deer and wild boar in two Austrian forest regions. J. Environ. Radioactiv..

[24] Ohtsuka-Ito E, Kanzaki N (1998). Population trends of the Japanese wild boar during the Showa era. Wildl. Cons. Jpn..

[25] Ueda H, Jiang Z (2004). The use of Orchards and Abandoned Orchard by wild boars in Yamanashi. Mamm. Sci..

[26] Fukushima Prefectural Government, Japan. Fukushima Prefecture Wild Boar Management Plan (Phase 3) (accessed 07 April 2021); https://www.pref.fukushima.lg.jp/uploaded/life/497785_1296285_misc.pdf (**in Japanese**).

[27] Anderson D (2019). A comparison of methods to derive aggregated transfer factors using wild boar data from the Fukushima Prefecture. J. Environ. Radioact..

[28] Pröhl G, Ehlken S, Fiedler I, Kirchner G, Klemt E, Zibold G (2006). Ecological half-lives of ^90^Sr and ^137^Cs in terrestrial and aquatic ecosystems. J. Environ. Radioactiv..

[29] Palo RT, White N, Danell K (2003). Spatial and temporal variations of ^137^Cs in moose *Alces alces* and transfer to man in northern Sweden. Wildlife Biol..

[30] Kodera Y, Kanzaki N, Ishikawa N, Minagawa A (2013). Food habits of wild boar (*Sus scrofa*) inhabiting Iwami District, Shimane Prefecture, western Japan. J. Mammal. Soc. Jpn..

[31] Kodera Y, Kanzaki N (2001). Food habits and nutritional condition of Japanese wild boar in Iwami district, Shimane Prefecture, western Japan. Wildl. Cons. Jpn..

[32] Arita S (2015). Radioactive cesium accumulation during developmental stages of Largemouth Bass, *Micropterus**salmoides*. Proc. JSCE. G. (Environment).

[33] Kodera YCSF (2019). prevention of epidemics from a point of view of the ecology of wild boar. J. Vet. Epidemiol..

[34] Calenge C, Maillard D, Vassant J, Brandt S (2002). Summer and hunting season home ranges of wild boar (*Sus scrofa*) in two habitats in France. Game Wildl. Sci..

[35] Massei G, Genov PV, Staines BW, Gorman ML (1997). Factors influencing home range and activity of wild boar (*Sus scrofa*) in a Mediterranean coastal area. J. Zool..

[36] Morelle K (2015). Towards understanding wild boar *Sus scrofa* movement: A synthetic movement ecology approach. Mammal Rev..

[37] Kapata J, Mnich K, Mnich S, Karpińska M, Bielawska A (2015). Time-dependence of ^137^Cs activity concentration in wild game meat in Knyszyn Primeval Forest (Poland). J. Environ. Radioactiv..

[38] Gulakov AV (2014). Accumulation and distribution of ^137^Cs and ^90^Sr in the body of the wild boar (*Sus scrofa*) found on the territory with radioactive. J. Environ. Radioactiv..

[39] Hohmann U, Huckschlag D (2005). Investigations on the radiocaesium contamination of wild boar (*Sus scrofa*) meat in Rhineland-Palatinate: A stomach content analysis. Eur. J. Wildl. Res..

[40] Škrkal J, Rulík P, Fantínová K, Mihalík J, Timková J (2015). Radiocaesium levels in game in the Czech Republic. J. Environ. Radioactiv..

[41] Japan Atomic Energy Agency (JAEA). 5th airborne monitoring survey (accessed 07 April 2021); https://emdb.jaea.go.jp/emdb/en/portals/b1020201/

[42] Steinhauser G (2017). Monitoring and radioecological characteristics of radiocaesium in Japanese beef after the Fukushima nuclear accident. J. Radioanal. Nucl. Chem..

[43] Merz S, Shozugawa K, Steinhauser G (2016). Effective and ecological half-lives of ^90^Sr and ^137^Cs observed in wheat and rice in Japan. J. Radioanal. Nucl. Chem..

